# P02.178. Skin conductance at 24 Source (Yuan) acupoints in 8637 patients: influence of age, gender and time of day

**DOI:** 10.1186/1472-6882-12-S1-P234

**Published:** 2012-06-12

**Authors:** S Chamberlin, A Colbert, A Larsen

**Affiliations:** 1 National College of Natural Medicine, Helfgott Research Institute, Portland, USA; 2 Meridia Technologies, Inc., Meridian, USA

## Purpose

The clinical practice of recording skin conductance (SC) at acupuncture points (acupoints), as a diagnostic and/or therapeutic monitoring aid may have scientific merit. However, influences of age, gender and time of day on these recordings are unknown and it is unclear whether SC at acupoints differs from SC levels in general (as reported in psychophysiology research). This research will investigate these influences.

## Methods

This analysis summarizes SC data obtained with the AcuGraph 3 Digital Meridian Imaging System between June 2005 and March 31, 2010. An initial dataset of 117,725 SC examinations was scrubbed to include only the first SC examination on individual patients and exclude potentially faulty data. The final dataset consists of SC recordings at the 24 Source (Yuan) acupoints in 8637 patients, collected by 311 practitioners. Twelve left/right average conductance measures and an overall average of the 24 acupoints were assessed. Statistical analyses included two sample t tests, three way analyses of variance and linear regression.

## Results

Results indicate that mean SC at acupoints, similar to SC in general, is higher in males, higher in afternoons and declines with age. Not previously reported, the rate of SC decline with age differs at different acupoints between males and females.

## Conclusion

These findings have substantial implications for acupuncture research and practice. Patterns derived from measures such as these should be investigated as potential early detectors of disease and predictors of treatment responsiveness.

